# Studies of Vietnamese pteridophyte flora 4: topotype project

**DOI:** 10.3897/phytokeys.275.194552

**Published:** 2026-05-27

**Authors:** Cheng-Wei Chen, Germinal Rouhan, Viet Dai Dang, Hong Truong Luu, Van Truong Do, Thi Ngan Lu, Yi-Shan Chao, Yao-Moan Huang, Kuo-Fang Chung

**Affiliations:** 1 Biodiversity Program, Taiwan International Graduate Program, Academia Sinica and National Taiwan Normal University, Taipei, Taiwan Taiwan Forestry Research Institute Taipei Taiwan; 2 Biodiversity Research Center, Academia Sinica, Taipei, Taiwan Vietnam National Museum of Nature, Vietnam Academy of Science and Technology Hanoi Vietnam; 3 Department of Life Science, National Taiwan Normal University, Taipei, Taiwan Institut de Systématique, Evolution, Biodiversité (ISYEB), Muséum national d’Histoire naturelle, CNRS, Sorbonne Université Paris France; 4 Institut de Systématique, Evolution, Biodiversité (ISYEB), Muséum national d’Histoire naturelle, CNRS, Sorbonne Université, EPHE-PSL, UA, Paris, France Biodiversity Program, Taiwan International Graduate Program, Academia Sinica and National Taiwan Normal University Taipei Taiwan; 5 Institute of Advanced Technology, Vietnam Academy of Science and Technology, Hochiminh, Vietnam Department of Life Science, National Taiwan Normal University Taipei Taiwan; 6 Vietnam National Museum of Nature, Vietnam Academy of Science and Technology, Hanoi, Vietnam Biodiversity Research Center, Academia Sinica Taipei Taiwan; 7 Taiwan Forestry Research Institute, Taipei, Taiwan Institute of Advanced Technology, Vietnam Academy of Science and Technology Hochiminh Vietnam

**Keywords:** Fern, flora, history, Indochina, systematics, taxonomy

## Abstract

Nomenclatural types serve as reference points in biodiversity research, providing a stable foundation for species naming and identification. Despite their critical role in taxonomy, these specimens are often from old collections and are inaccessible for study using modern methodologies, such as molecular phylogenetics and reproductive biology. This gap can be bridged by recollecting new topotypic materials from type localities. In 2025, the “Vietnamese Pteridophyte Topotype Project” was launched using this approach to advance systematics studies. Here, the first results of this project are presented, including a detailed analysis of Vietnamese pteridophyte types, with emphasis on their historical background, taxonomic composition, and systematic significance. In addition, lectotypes are designated for *Pteris quadriaurita* var. parviloba Christ and *Trichomanes javanicum* var. *glabra* Bonap.

## Introduction

In biology, a “type” is an element (usually a specimen) to which the scientific name of an organism is permanently linked (Turland et al. 2025, Article 7.2), ensuring stability and clarity in species identification. It thus plays a central role in nomenclature and systematics. Modern systematics increasingly emphasizes integrated approaches, including phylogeography, comparative morphology, population genetics, and reproductive biology (Dayrat 2005). However, many historical type specimens are unsuitable for these methods. For example, chromosome counts require fresh material, and the absence of usable type specimens constrains contemporary research and conservation efforts, especially when species boundaries are uncertain (Stuart and Fritz 2008). A practical solution is to collect new specimens from type localities that serve as topotypic vouchers, as demonstrated by Bell et al. (2020). Such materials enable the application of modern methods and support more comprehensive systematic studies.

Pteridophytes—ferns and lycophytes—comprise nearly 12,000 species worldwide (PPG 2016) and are particularly diverse in the tropical forests of Southeast Asia, including Vietnam. The first records of Vietnamese pteridophytes date to the 18^th^ century, but large-scale collections began only during the French colonial period (1858–1954), which greatly expanded knowledge of the flora (Chen et al. 2021). This period also saw the publication of the first comprehensive pteridophyte flora (Tardieu-Blot and Christensen 1939–1951). Progress was later interrupted by wars (1946–1954, 1955–1975) and subsequent economic difficulties. Since the 1990s, however, Vietnam’s economic recovery and renewed international collaborations have triggered a second wave of species discovery that continues today (Chen et al. 2021). Over the past 2 decades, local and international botanists have accumulated extensive new data, especially through new field expeditions (e.g., Wu et al. 2005; Parris et al. 2015; Li et al. 2024). While these studies have advanced knowledge primarily by describing new species and reporting new records, they have rarely re-examined poorly known species—many represented only by type specimens—described during the colonial era.

To address this gap, the “Vietnamese Pteridophyte Topotype Project” was launched in April 2025, aiming to revisit type localities and collect topotypic vouchers to support modern systematic studies. As an initial step, the first author spent 3 months working at the herbarium P (codes follow Thiers 2026) of the Muséum national d’Histoire naturelle (MNHN) in Paris, where most Vietnamese type specimens from the colonial period are deposited. Here, part of the results are presented, including: (1) an overview of Vietnamese pteridophyte type specimens and type localities, with emphasis on those deposited at P; (2) a review of the principal collectors of Vietnamese pteridophyte types; and (3) a discussion of the systematic implications of newly identified types. Through selected case studies, these findings demonstrate how they contribute to a clearer understanding of Vietnam’s pteridophyte diversity.

## Materials and methods

The checklist compiled by Chen et al. (2021), which includes 222 names of Vietnamese type specimens, was used as the starting point. This list was expanded through several steps. First, the original protologues of all names were examined, and voucher information was extracted, including type localities, collectors, collection numbers, herbaria of deposit, year of collection, and year of publication. Next, relevant historical literature was reviewed using resources such as the Biodiversity Heritage Library (https://www.biodiversitylibrary.org/) and the library of the MNHN. Google Scholar (https://scholar.google.com/) was also searched using keywords such as “fern,” “lycophyte,” “Vietnam,” and “new species” to identify names published after Chen et al. (2021). In addition, JSTOR (https://plants.jstor.org/) and PteridoPortal (https://www.pteridoportal.org/portal/index.php) were consulted to identify type specimens not deposited in P.

Fieldwork was conducted to revisit the type localities in 2025 and 2026. While compiling locality data, many original type localities were found to lack clear modern equivalents, likely due to name changes, exonyms, or transcription errors. In such cases, early 20^th^-century maps (e.g., de Chabert and Gallois 1909) and other historical references (e.g., Tardieu-Blot 1932) were consulted. Where the original locality remained ambiguous, possible sites were inferred based on species’ ecological preferences, or biographical information of the collectors was consulted (e.g., Cadière 1906; Chevalier 1942). In many instances, substantial landscape changes over recent decades have altered the habitats of type localities. For these cases, the nearest national parks or protected areas were identified as proxies. To avoid confusion, all locality names are presented in Vietnamese throughout the article. To illustrate the geographic distribution of type localities, the interpreted sites were georeferenced using GeoPick v.2.1.1 (Marcer et al. 2024), following the best practices of Chapman and Wieczorek (2020), and the results were visualized in R (R Core Team 2025).

The biographical information of five major collectors of Vietnamese types was collected from biographical studies (e.g., Chevalier 1942; Burgos and Carré 2021), websites (e.g., Dartigues 2018; IRFA 2025), their own publications (e.g., Balansa 1888; Cadière 1906; Eberhardt 1927, 1931; Pételot and Magalon 1929), and their voucher specimens deposited in P.

## Results and discussion

### Vietnamese pteridophyte collections in Paris and their collectors

In this study, the number of recognized Vietnamese pteridophyte types was increased from 222 (Chen et al. 2021) to 250 (Suppl. material 1). The types of 194 names (77.6%) were located at P. Of the 250 names documented, 185 types (74%) were collected by French botanists between 1837 and 1937. Although P is widely acknowledged as housing the most extensive and comprehensive collection of Vietnamese pteridophytes, no clear estimate exists of the total number of specimens. Since the launch of its digitization initiative in 2008 (Le Bras et al. 2017), the herbarium has made considerable efforts to document and digitize its holdings. Nevertheless, most Vietnamese pteridophyte specimens remain only partially databased, typically including only identifications, barcode numbers, and broad geographic information. Consequently, it remains difficult to accurately quantify the extent of the relevant collections. Future work should aim to systematically identify and fully database all Vietnamese pteridophyte specimens. Only with such data can key questions be addressed, including the following: How many collections were made? Who collected them? When and where were the expeditions conducted? These data would also enable more refined analyses, such as comparing historical and present-day species distributions to evaluate land-use changes over time. Another critical issue is the need for lectotypification. Many names were published based on multiple specimens without a designated type. However, such typification is best undertaken in the context of comprehensive taxonomic revisions and, therefore, was not attempted here, except for two cases (see the “Newly found Vietnamese pteridophyte types” section).

**Figure 1.**
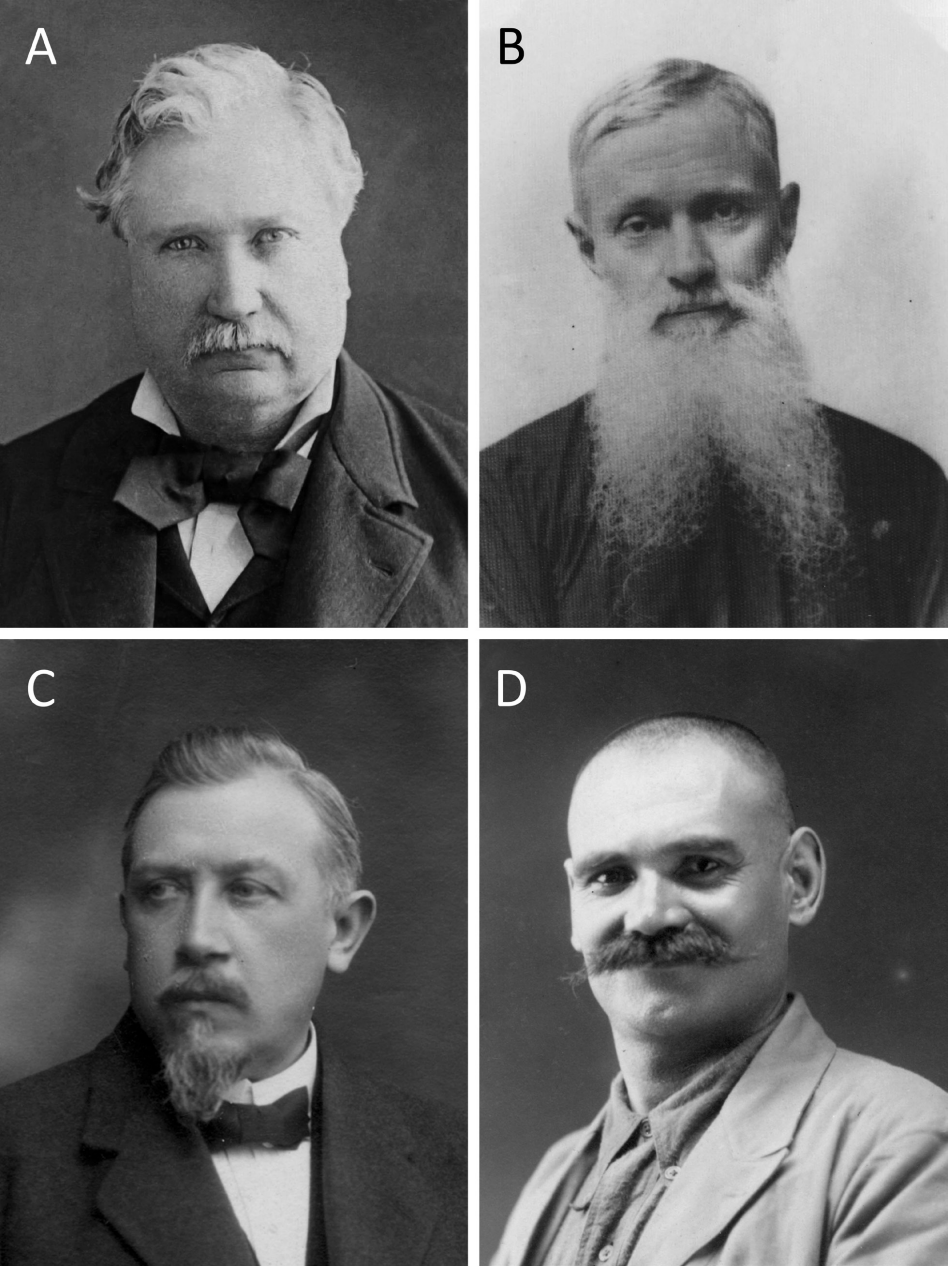
Portraits of four major French collectors of Vietnamese pteridophyte types. **A**. Benjamin Balansa. Portrait by Jules de Lacger, 2 May 1885. Original photograph deposited in the Toulouse Museum; **B**. Léopold Cadière. (https://irfa.paris/en/missionnaire/2034-cadiere-leopold/); **C**. Philippe Albert Eberhardt. Portrait sent by him on 3 May 1921 to the MNHN; **D**. Eugène Poilane. Portrait sent by him in early 1926 to the MNHN.

In the following paragraphs, the contributions of the five most prolific collectors of Vietnamese pteridophyte types are reviewed (Fig. 1). It should be noted that this review is based solely on type specimens and may not capture the full geographic scope of their expeditions in Vietnam.

#### Benjamin Balansa (1825–1891)

(Fig. 1A)

*“Alone, unarmed, and never disturbed, this gentle old man wandered through the bush, respected by all. The Tonkinese had nicknamed him the Western old man. He had only one passion: botany; and only one ambition: to make it useful.”*

Balansa was a prolific French naturalist whose lifelong work yielded more than 20,000 herbarium specimens. His career, thoroughly reviewed by Chevalier (1942), spanned expeditions to North Africa (Algeria, Morocco), the Middle East (Turkey), South America (Paraguay), the Pacific Islands (New Caledonia), and Asia (Vietnam). From 1885 to 1888, he conducted extensive fieldwork in Tonkin (northern Vietnam), with a particular focus on Mount Ba Vì (1,296 m). In one letter, he wrote: “*The flora of this country is truly inexhaustible; I’ve been exploring Mount Bavi for over a year, and I’m far from having discovered everything. In none of my previous trips have I seen such a diversity. For an equal area, Tonkin is certainly the country in the world with the richest flora, especially considering the low height of its mountains*” (Chevalier 1942). Of the 31 Vietnamese pteridophyte names described from his collections, 22 were collected from Ba Vì. His expedition in Vietnam was a remarkable achievement, yielding more than 5,600 specimens—an especially impressive feat considering that he began this work at the age of 60. Balansa was not merely a collector; he also published on his observations, with particular interest in grasses, describing new species and producing local floristic lists (Balansa 1890). Notably, he authored the first account of the geography and vegetation of Tonkin (Balansa 1888).

#### Léopold Cadière (1869–1955)

(Fig. 1B)

*“Learning a language is not merely a matter of the throat or the ear; it is not merely a matter of memory. When it comes to languages as different as French and Annamite, it is above all a matter of thought. It is not enough to speak like the Annamites — one must also think as they do.”*

Cadière was a French missionary, historian, and anthropologist who arrived in Huế, central Vietnam, in 1892 and spent most of his life in the region. Best known for his extensive publications on the history, religion, and language of Vietnam (Dartigues 2018), he is widely regarded as one of the founders of ethnological studies in the country. He was also among the founders of the *Bulletin des Amis du Vieux Hué*, a journal that covered a wide range of subjects reflecting Cadière’s broad interests, including history, geography, ethnology, fine arts, literature, and religion. Although less known for his botanical work, Cadière was an active plant collector whose thousands of specimens are now deposited in P. His collections include 23 Vietnamese pteridophyte taxa described from his material. Notably, he was the first to conduct extensive botanical collecting in Quảng Bình and Quảng Trị provinces—regions previously unexplored for their flora. Part of his collections from these areas were studied by Christ (1905), who enumerated 123 species of pteridophytes. Cadière appeared to have a particular fascination with ferns, publishing a work on the ferns of Quảng Bình Province (Cadière 1906) and establishing a botanical garden renowned for its collection of rare fern species (IRFA 2025).

#### Philippe Albert Eberhardt (1874–1942)

(Fig. 1C)

Eberhardt, a doctor of natural sciences born in Paris, arrived in Vietnam in 1905 on a scientific mission. Eberhardt devoted much of his career to the study of the flora and agricultural resources of Indochina. He sought to link pure botanical research with the economic needs of the colony, particularly in the domains of tropical crops and the utilization of local plants. Actively involved in scientific education and outreach, he also authored botanical and zoological textbooks for schools and published works on botany, agriculture, and forestry (e.g., Eberhardt 1927, 1931). Through these works, he played a decisive role in the dissemination of scientific knowledge in early 20^th^-century Vietnam. In recognition of his scientific merits, he was appointed as tutor to the young king Duy Tân of Annam from 1909 to 1916. He was the first to carry out systematic botanical surveys of Tam Đảo and the Lang Biang Plateau—the latter now recognized as a biodiversity hotspot (Luu et al. 2023). Christ (1908) recorded 98 pteridophyte species from Eberhardt’s collections from these areas, including eight species newly described at the time. In total, 33 Vietnamese pteridophyte taxa were established from his material, marking his work as a major contribution to the country’s fern flora.

#### Paul Alfred Pételot (1885–1965)

*“Look at the plants around you, study them in detail, collect and dry them so that you may keep them before your eyes for longer; in this way, you will learn botany effortlessly, with pleasure and with benefit.”*

Pételot was a French botanist and entomologist who worked extensively in the former French colonies of Indochina, devoting much of his research to the medicinal plants of the region. He served as a lecturer at the Mixed Faculty of Medicine and Pharmacy in Saigon and later headed the botanical division of the Scientific and Technical Research Center, where he also coauthored a botany textbook (Pételot and Magalon 1929). Although more than 150 plant species have been named in his honor, information on his substantial body of work remains fragmentary, and no comprehensive bibliographic study of his contributions has yet been undertaken. Nineteen pteridophyte names were described from his Vietnamese collections, most of which were made in the northern part of the country. He was particularly active in the Hoàng Liên mountain range, home to Vietnam’s highest peak (Hoàng Liên Sơn, 3,143 m), where he assembled a large and taxonomically important fern collection.

#### Eugène Poilane (1887–1964)

(Fig. 1D)

“*It was to gather materials, to carry out botanical inventories, that I traveled across Indochina*.”

Poilane was an exceptional naturalist whose work represents perhaps the most extensive botanical collecting effort in Vietnam. Arriving at the age of 21, he was trained by renowned botanist Auguste Chevalier and later devoted his life to botanical inventory across the country. As he once wrote: “*I am no longer French, but Indochinese*.” His life and contributions, recently reviewed in detail by Burgos and Carré (2021), extended far beyond herbarium specimens to include collections of wood, minerals, reptiles, fishes, amphibians, and insects. Twenty Vietnamese pteridophyte names were based on his collections, several of which—such as *Colysis poilanei* C.Chr. & Tardieu, *Dryopteris poilanei* Tardieu, and *Tectaria poilanei* Tardieu—were known only from material he gathered. Poilane explored many remote, little-surveyed regions, such as Lai Châu, Ninh Thuận, and Quảng Nam provinces, underscoring his commitment to documenting Vietnam’s biodiversity.

### Type localities with clarification of some old names

**Figure 2.**
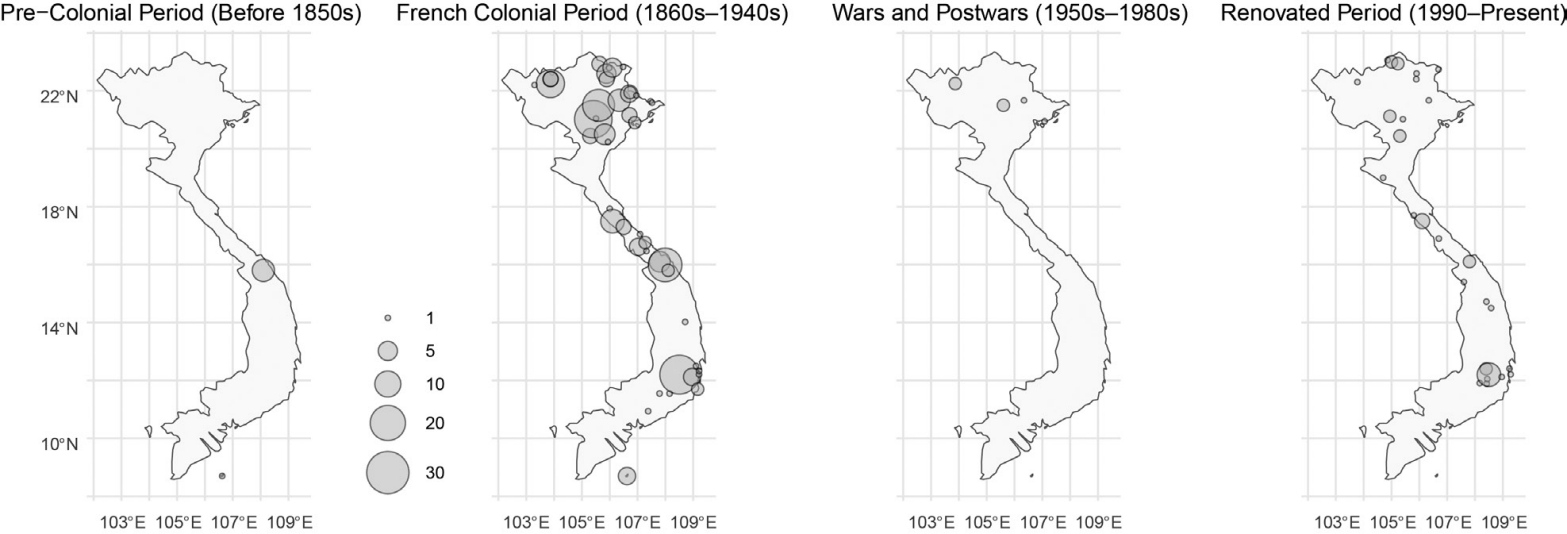
Vietnam map showing the type localities of 250 Vietnamese pteridophyte types, grouped into four periods following Chen et al. based on collection year. Circle size indicates the number of names published. The Trường Sa and Hoàng Sa Islands are not shown, as no type specimens have been collected there.

Fig. 2 illustrates the type localities of 250 Vietnamese pteridophyte names, categorized into four historical periods defined by Chen et al. (2021) based on the year of collection. Prior to the 1850s, only eight type specimens were collected, seven of which were made by Charles Gaudichaud-Beaupré in Đà Nẵng—then one of the most important international trading ports in Vietnam. During the French colonial period, French botanists conducted extensive botanical exploration throughout the country, particularly in Tonkin (northern Vietnam), resulting in many pteridophyte types. In contrast, only six types were collected during the wars and postwar period, all by Vietnamese botanists and exclusively from northern Vietnam. In the past 2 decades, a second wave of species discovery has emerged, driven by Vietnam’s economic development and increased international collaboration. These recent collections include not only new discoveries from historically well-studied areas but also specimens from previously underexplored regions, such as *Cyrtomium elongatum* S.K.Wu & P.K.Lôc from Hà Giang (now Tuyên Quang Province) and *Arachniodes longicaudata* Li Bing Zhang, N.T.Lu & Liang Zhang from Nghệ An Province. Fig. 2 also highlights several apparent “gaps” on the map, such as Điện Biên Province, Gia Lai Province, and the Mekong Delta. Further fieldwork is needed to determine whether these gaps reflect true patterns of lower pteridophyte diversity or simply result from limited botanical exploration.

While compiling the type locality data, several historical place names that lack clear modern equivalents were encountered. In the following paragraphs, the modern identities of three of these historical localities are clarified.

Balansa made abundant collections from the locality labeled “Tonkin, Tu Phap.” A search in the JSTOR Global Plants database reveals over 300 specimens attributed to Balansa from this area. According to Chevalier (1942), Balansa traveled along the Sông Hồng (Red River) and then into the Sông Đà (Black River), eventually reaching Tu Phap. One of his specimens (P00744419) includes the note: “Bois près de la rive gauche de la Rivière Noire en face de Tu Phap” (Woods near the left bank of the Black River, opposite Tu Phap). The French colonial map of Tonkin (de Chabert and Gallois 1909) marks Tu Phap as a village situated along the Black River, which is also known as Thủ Pháp, located in Bất Bạt District, Sơn Tây Province (Pham 1941). Historically, this area was covered by dense forest, but it has since been extensively transformed by agriculture and human settlement. As a result, the western slope of the Ba Vì mountain range—near the historical Tu Phap locality and still supporting suitable forest habitats—should serve as a topotypic substitute for specimens originally labeled from “Tu Phap.”

Tourane is the old French colonial name for what is now Đà Nẵng, a major port city in central Vietnam. Charles Gaudichaud-Beaupré (1789–1854), who traveled with the La Bonite expedition in 1836–1837, visited Tourane from 24 January to 5 February 1837 (Léandri 1971). A search for “Tourane” in the JSTOR Global Plants database reveals over 100 plant specimens collected by Gaudichaud-Beaupré from this locality. Among these, six ferns were later published based on his collections: *Lomariopsis cochinchinensis* Fée, *Lacaussadea montana* Gaudich., *Hemicardion crenatum* Fée, *Aspidium trichotomum* Fée, *Angiopteris cochinchinensis* de Vriese, and *Drymoglossum abbreviatum* Fée. Based on field observations, most of these species are typically found in low- to mid-elevation primary forests with stable humidity—habitats that no longer exist within the modern urban landscape of Đà Nẵng. Nearby remnants of lowland forest, such as those on the Sơn Trà Peninsula or in the Bà Nà Hills, therefore represent the most ecologically appropriate topotypic substitutes for Gaudichaud’s Tourane collections.

Wai-Tak Tsang, a Chinese plant collector affiliated with Lingnan University in Hong Kong, made an extensive botanical survey along the Chinese border with Vietnam from 1937 to 1940. He collected approximately 2,000 numbers of specimens from that area (Li 1944), and many were labeled from “Taai Wong Mo Shan.” This name is clearly not Vietnamese, but rather a Cantonese romanization of the Chinese name “大黃毛山,” which translates to “Big Yellow Hair Mountain.” It is likely that this translation was made by Tsang himself, as it could not be found elsewhere except on his specimen labels. A mountainous commune near the China–Vietnam border called “Hoành Mô,” located in Quảng Ninh Province, was noted. The name “Hoàng Mô” literally means “Yellow Hair,” suggesting a direct translation match. Based on this linguistic and geographic evidence, “Taai Wong Mo Shan” corresponds to the present-day Hoành Mô Commune in Quảng Ninh Province, Vietnam.

### Newly found Vietnamese pteridophyte types

Among the 194 names with types deposited at P, the types of 26 names—eight at the species rank and 18 at the infraspecific rank—that had not been previously recognized elsewhere were newly located (Table 1). Although some of these names had been cited in earlier taxonomic works, the whereabouts of their original material were either unclear or entirely undocumented. In the following paragraphs, four names that are tentatively treated as distinct species are discussed: *Pteris parviloba* (Christ) Christ, *P. quadriaurita* Retz. var. *infurcata* Bonap., *Polystichum tonkinense* (Christ) W.M.Chu & Z.R.He, and *Asplenium eberhardtii* Tardieu. Two additional names that are treated as synonyms are also discussed: *Trichomanes javanicum* Blume var. *glabra* Bonap. and *Aspidium distans* (D.Don) Christ var. *cadieri* Christ. For the remaining 20 names, which are tentatively treated as synonyms, most were originally described at the infraspecific rank and differ only slightly from the typical variety. Further evidence—morphological, molecular, or ecological—is needed to assess whether any of these taxa warrant recognition as distinct entities.

**Table 1.** Twenty-six names for which the types were newly identified in this study, along with their provisional identifications based on preliminary morphological comparisons. The table is arranged alphabetically by basionym. Names discussed further in the text are indicated in bold. Voucher information for all basionyms is available in Suppl. material 1.

Basionyms	Provisional accepted name
*Angiopteris repandula* de Vriese var. *latemarginata* Ching ex C.Chr. & Tardieu	*Angiopteris dianyuecola* Z.R.He & W.M.Chu
***Aspidium aculeatum* Sw. var. tonkinense Christ**	***Polystichum tonkinense* (Christ) W.M.Chu & Z.R.He**
***Aspidium distans* (D.Don) Christ var. *cadieri* Christ**	***Tectaria kusukusensis* (Hayata) Lellinger**
*Asplenium annamense* Christ	*Asplenium scortechinii* Bedd.
***Asplenium eberhardtii* Tardieu**	***Asplenium eberhardtii* Tardieu**
*Asplenium nephrolepioides* Christ	*Asplenium normale* D.Don
*Asplenium tenerum* G.Forst. var. *stenophyllum* Bonap.	*Asplenium thunbergii* Kunze
*Athyrium pseudosetigerum* Christ var. *bonii* Tardieu	*Diplazium pseudosetigerum* (Christ) Fraser-Jenk.
*Blechnum orientale* L. var. *bipinnatum* Bonap.	*Blechnopsis orientalis* (L.) C.Presl
*Cyclophorus induratus* Christ	*Pyrrosia longifolia* (Burm.fil.) C.V.Morton
*Davallia strigosa* (Thunb.) Sw. var. *subciliata* Christ	*Microlepia marginata* (Panz.) C.Chr.
*Diplazium aridum* Christ	*Diplazium doederleinii* (Luerss.) Makino
*Diplazium esculentum* (Retz.) Sw. f. *squamulosa* Bonap.	*Diplazium esculentum* (Retz.) Sw.
*Diplazium megaphyllum* (Baker) Christ var. *subintegrifolium* Tardieu	*Diplazium megaphyllum* (Baker) Christ
*Diplazium stoliczkae* Bedd. f. *longipinnulatum* Bonap.	*Diplazium stoliczkae* Bedd.
*Diplazium stoliczkae* Bedd. f. *brevipinnulatum* Bonap.	*Diplazium stoliczkae* Bedd.
*Drynaria mutilata* Christ	*Drynaria parishii* (Bedd.) Bedd.
*Dryopteris parasitica* (L.) Kuntze var. *aureoglandulosum* Bonap.	*Christella parasitica* (L.) H.Lév.
*Dryopteris subconjuncta* Christ	*Alsophila podophylla* Hook.
*Nephrolepis hirsutula* (G.Forst.) C.Presl var. *rotundatipinnata* Bonap.	*Nephrolepis hirsutula* (G.Forst.) C.Presl
*Pteris indochinensis* Christ	*Pteris insignis* Mett. ex Kuhn
***Pteris quadriaurita* Retz. var. *infurcata* Bonap**.	***Pteris*** sp.
***Pteris quadriaurita* Retz. var. parviloba Christ**	***Pteris parviloba* (Christ) Christ**
*Pteris vittata* L. var. *latebasis* C.Chr.	*Pteris vittata* L.
***Trichomanes javanicum* Blume var. *glabra* Bonap**.	***Cephalomanes sumatranum* (Alderw.) Copel**.
*Trichomanes rigidum* Sw. var. *platyrachis* Christ	*Vandenboschia cystoseiroides* (Christ ex Tardieu & C.Chr.) Ching

*Pteris parviloba* was demonstrated to be a morphologically and phylogenetically distinct species by Chao et al. (2017), who clarified its differences from the similar *P. decrescens* Christ. Although their taxonomic treatment is accepted, two issues were noted in their typification. First, Chao et al. (2017) incorrectly attributed the authorship of the name *P. parviloba* to Christ. However, the epithet parviloba was first published by Christ and Billet (1898: 264) as a variety of *P. quadriaurita* Retz. and later elevated by Christ himself to species rank (Christ 1907: 149). Therefore, the correct authorship is “(Christ) Christ.” Second, Chao et al. (2017) designated Esquirol’s specimen (*no. 712*, from China) as the lectotype, but it was not cited in the protologue. Instead, Christ and Billet (1898) clearly stated in the introduction that the species was based on material collected by Billet from Cao Bằng, Vietnam. Two such specimens were found in P: one collected in 1892 (P01291322) and the other in 1896 (P01291306). As the former matches the protologue in both date and locality, it is designated as the lectotype:

***Pteris quadriaurita* var. parviloba Christ, Bull. Sci. France et Belgique 28: 264, pl. XII, fig. 3. 1898**

**Lectotype (here designated)**. Vietnam, Cao Bằng, Quảng Uyên, 16 March 1892, *A. Billet s.n*. (P! [P01291322]).

**Currently accepted name**. *Pteris parviloba* (Christ) Christ, Bull. Acad. Int. Géogr. Bot. sér. 3, 17(212): 149. 1907.

*Pteris quadriaurita* Retz. var. *infurcata* was described by Bonaparte (1918: 180) based on Eberhardt’s collection (*no. 390*) from the Cu-Bi River, located north of Huế. While this locality has no modern equivalent, it appears on de Chabert and Gallois’s 1909 map and is now the site of the Hương Điền Hydroelectric Dam. A single specimen was identified at P (P01298314) that matches the protologue and bears Bonaparte’s annotation, and it is therefore recognized as the holotype. The specimen contains three individuals, all juvenile and sterile, making it difficult to assess species-level distinctiveness. Fortunately, during a recent expedition to Quảng Nam Province, approximately 150 km south of the type locality, fertile specimens of the same taxon were collected. These mature individuals are morphologically distinct from all currently known *Pteris* species in Vietnam, characterized by large plants exceeding 1 m in height, 1-pinnate-pinnatifid fronds with up to three pairs of pinnae, and broad pinnae up to 5 cm wide. *Pteris quadriaurita* is an American species that has been widely misapplied in Asia. Although it is clear that *P. quadriaurita* var. *infurcata* represents a species distinct from *P. quadriaurita*, morphologically similar taxa have been reported from neighboring regions, such as *P. longipinnula* Wall. ex J. Agardh and *P. clemensiae* Copel. Further study is required to clarify its systematic position.

*Polystichum tonkinense* was originally described by Christ and Billet (1898: 268) as a variety of *Aspidium aculeatum* Sw., based on Billet’s collection (*s.n*.) from limestone mountains in Cao Bằng Province near the border with Guangxi, China. Chu and He (2001) later raised it to species rank in their revision of the Chinese flora, although they did not indicate whether the original material was examined. A single specimen was located at P (P01351505) that matches the protologue and bears Christ’s handwriting and illustration, and it is recognized as the holotype. While the specimen is poorly preserved, it shows key diagnostic features, including an ascending rhizome, scales on the stipes and rachis, elliptic-lanceolate fronds, and bipinnate laminae with small pinnules (up to 2 cm long).

*Asplenium eberhardtii* was described by Tardieu-Blot (1933: 484) based on Eberhardt’s collection (*no. 1888*) from Lang Biang Mountain. A single specimen was identified at P (P01398335) bearing Tardieu-Blot’s annotation that matches the protologue, and it is recognized as the holotype. Morphologically, this species is part of a historically confusing complex often misidentified as *A. affine* Sw. (type: Mauritius, Viane 2021), *A. cuneatum* Lam. (type: probably Jamaica, Cremers and Viane 2008), or *A. spathulinum* J.Sm. ex Kunze (type: Philippines, Sledge 1962). *Asplenium eberhardtii* is tentatively accepted as a distinct species, but the need for detailed comparative studies, especially with similar taxa from Asia, to clarify its systematic position is emphasized.

*Trichomanes javanicum* var. *glabra*, described by Bonaparte (1918: 142), belongs to the genus *Cephalomanes*, which was recently revised by Zhao et al. (2025). According to their study, *Cephalomanes javanicum* (Blume) C.Presl and *C. sumatranum* (Alderw.) Copel. are both present in Vietnam. Although Bonaparte’s variety was not mentioned by Zhao et al. (2025), it clearly falls within this group. Bonaparte cited three syntypes in the protologue, all of which were located at P with his annotations (P01323054, P01323071, P01323072). All of these specimens are morphologically consistent with *C. sumatranum* following Zhao et al. (2025). Cadière’s collection (P01323054) is designated because it consists of a single individual with abundant fertile fronds:

***Trichomanes javanicum* var. *glabra* Bonap., Notes Pteridol. 7: 142. 1918**

**Lectotype (here designated)**. Vietnam, Huế, Thanh Tân, February 1905, *Cadière 3 [116]* (P! [P01323054]).

**Currently accepted name**. *Cephalomanes sumatranum* (Alderw.) Copel., Philipp. J. Sci. 67: 67. 1938.

**Note**. Bonaparte (1918: 142) mistakenly cited Cadière’s collection as originating from Ba Lòng, Quảng Trị.

*Aspidium distans* var. *cadieri* was described by Christ (1905: 63) based on Cadière’s collection (*no. 91*) from Annam (central Vietnam). Tardieu-Blot and Christensen (1939–1951) later synonymized the taxon under *Glaphyropteridopsis erubescens* (Wall. ex Hook.) Ching (Thelypteridaceae). In this study, two specimens were found at P labeled as Cadière *no. 91* (P01452387 and P01575750), both of which clearly belong to *Tectaria kusukusensis* (Hayata) Lellinger (Tectariaceae). Notably, P01452387 bears an annotation by Tardieu-Blot identifying it as *T. kusukusensis* and was cited in Tardieu-Blot and Christensen (1938), indicating that she was familiar with this species and unlikely to have misidentified it as *G. erubescens*. The second specimen, although labeled “Aspidium distans var. cadieri,” does not bear Christ’s handwriting. Additionally, many of Cadière’s specimens carry dual numbers; in this case, *20 [91]*, suggesting that additional specimens under the same number might exist. However, after thorough examination of all Vietnamese Thelypteridaceae collections at P, no other material matching that number was found.

### Côn Đảo, the origin of Vietnamese pteridology

*Schizaea dichotoma* (L.) Sm. (basionym *Acrostichum dichotomum* L., 1753) is the first pteridophyte described from Vietnam (see below). This name has since been widely applied across the Old World tropics and subtropics—from East Africa, Madagascar, and the Mascarene Islands to southern India, Sri Lanka, Southeast Asia, Australia, New Zealand, and throughout the Pacific from New Caledonia to the Marquesas Islands (Brownsey and Perrie 2014). However, molecular phylogenetic analysis by Ke et al. (2022) revealed that *S. dichotoma* is polyphyletic, with populations resolving into two distinct clades: one comprising Asian/Oceanian taxa and another consisting of African, Madagascan, and Mascarene Island populations. Since the type of *S. dichotoma* originates from Asia, the African, Madagascan, and Mascarene populations were subsequently described as a separate species, *S. medusa* L.Y.Kuo, B.F.Ke, F.W.Li & Rouhan.

Nevertheless, even within the Asian/Oceanian clade, specimens of *Schizaea dichotoma* remain polyphyletic, indicating that further study is needed to resolve species boundaries within this complex. Adding to the complexity is the confusion surrounding the exact type locality of *S. dichotoma*. When Linnaeus (1753: 1068) described the species based on Petiver’s illustration (*“Filix cochine. Pet. gaz. t. 70. f. 12”*), he mistakenly recorded China as the type locality. Dandy and Britten (1958) were the first to point out that the correct locality is Cochinchina (an exonym for present-day southern Vietnam in the early 18^th^ century), based on material collected by the Scottish physician and botanist James Cunninghame. Petiver’s illustration, upon which Linnaeus based the name, was selected as the lectotype by Holttum (1959: 41). A specimen in Sloane’s herbarium at BM, which matches this illustration and was collected by Cunninghame, should be recognized as a typotype (Prado et al. 2022). Cunninghame was employed as a surgeon by the English East India Company and worked at Pulo Condore (now Côn Đảo) in 1703 (Tze-Ken 2012). Unfortunately, he was later captured and put on trial in Barrea (modern Bà Rịa) following a mutiny for 2 years. He was eventually either released or escaped and made his way to Borneo and never returned to Vietnam.

Based on this historical context, Côn Đảo is proposed as the most likely type locality of *Schizaea dichotoma*. This hypothesis is further supported by the later collection of this species from Côn Đảo, *Harmand 707* (VNM00009843). To clarify the systematics of this species complex, future studies should include samples from Côn Đảo. In 2025 and 2026, two week-long field expeditions were conducted in Côn Đảo in search of this species. A wide range of habitats was surveyed, including coastal forests, rainforests, and riversides, reaching elevations of nearly 550 m (the island’s summit is 577 m), but the species was not found. Possible explanations include extremely small population sizes or local extinction; however, more extensive field investigations will be necessary to clarify this in the future.

### Bà Nà, a testament of biodiversity loss in Vietnam

Located ca. 40 km west of the city of Đà Nẵng in central Vietnam, Bà Nà–Núi Chúa special-use forest, with its iconic Bà Nà hill, is one of the most popular tourism destinations for both domestic and international travelers (VietnamPlus 2024). Bà Nà hill is located nearly 1,500 m above sea level, and a tourism station was first founded in 1919 by French colonists because of its cooler weather. Bà Nà hill was rich in biodiversity resources, with at least six primate species, more than 50 species of snakes, 49 species of termites, and 280 species of medicinal plants (Pham et al. 2012; H.T. Pham et al. 2015; T.K.T. Pham et al. 2015; Bui et al. 2019).

The French tourism station was destroyed during the war (1946–1954) and abandoned. In 1997, the local government developed a project to rebuild Bà Nà into an ecotourism area but did not achieve economic results. In 2007, the project was operated by Sun Group. Currently, the summit of Bà Nà hill has been completely transformed into a resort complex that covers more than 200 hectares, and it represents a successful story in terms of economy and tourism, as presented in official and mainstream media. However, the environmental impact of such large-scale construction has rarely been considered and discussed. A rare exception is the newspaper *Phụ nữ*, which published a series of reports raising concern over deforestation at Bà Nà (Phu Nu 2019a, b, c, d) but was eventually fined (Nhan Dan 2020). Here, using pteridophytes as an example, further evidence of biodiversity loss is provided, and many species may have become extinct either locally or even globally due to deforestation.

Soon after the resort was established in 1911, numerous botanical collections were made between 1920 and 1934, leading to the description of 16 new pteridophyte taxa. To recollect new topotypic materials, a 4-day expedition was conducted in August 2025, exploring various forest types across different elevations. Several species newly recorded in the country and a potentially undescribed species were collected and will be reported in a separate paper, indicating that primary forest areas had been reached. Unfortunately, only eight species were ultimately located, and the missing ones are *Arachniodes tonkinensis* (Ching) Ching, *Blechnum orientale* L. var. *bipinnatum* Bonap., *Macrothelypteris banaensis* (Tardieu & C.Chr.) Christenh., *Microlepia herbacea* Ching & C.Chr. ex Tardieu & C.Chr., *Nephrolepis hirsutula* (G.Forst.) C.Presl var. *rotundatipinnata* Bonap., *Phlegmariurus obovalifolius* (Bonap.) A.R.Field & Testo, *Selaginella rolandi-principis* Alston, and *Selliguea banaensis* (C.Chr.) B.-Y.Sun & I.C.Hwang. There are different possible explanations for these missing taxa. For *Blechnum orientale* L. var. *bipinnatum*, it is simply a crested individual with pinnatifid pinnae (rather than entire, as in normal individuals) of a common species. Fasciation has been widely reported across many vascular plants but is generally rare (Iliev and Kitin 2011). Similarly, *Nephrolepis hirsutula* (G.Forst.) C.Presl var. *rotundatipinnata* might be a dwarf and occasional form growing in exposed sites and therefore much smaller than the typical form. *Phlegmariurus obovalifolius* was reported from Bà Nà a decade ago (Duy et al. 2016), suggesting that it might still be present there in small populations, as species of the genus are generally rare in the field. *Arachniodes tonkinensis*, *Selliguea banaensis*, *Selaginella rolandi-principis*, and *Microlepia herbacea* are more widespread in Vietnam and are usually found at higher elevations, such as the Lang Biang Plateau at the southern end of the Annamite range. In Bà Nà, these species were most likely restricted to the mossy forests near the summit, which are now entirely devoid of natural habitat and may have led to their local extinction there. *Macrothelypteris banaensis* is a poorly known species represented solely by the type collection. The genus itself is relatively small, comprising only nine species worldwide (Holttum 1969), and *M. banaensis* can be readily distinguished from its congeners by its oblong fronds and scaly rachis. Further field expeditions in Bà Nà and the surrounding mountain ranges are needed to confirm whether the species still persists.

## Additional information

### Artificial Intelligence (AI) use

The authors accept full responsibility for the content of the manuscript, including the disclosure of any use of AI.

The following AI tools were used in the preparation of this manuscript:

**AI tools**: GPT.

**Used for**: Language, style and writing.

### Author ORCIDs

C.-W. Chen https://orcid.org/0000-0001-9709-6739

G. Rouhan https://orcid.org/0000-0002-0751-5352

V.D. Dang https://orcid.org/0000-0002-3462-9419

H.T. Luu https://orcid.org/0000-0002-7036-7081

V.T. Do https://orcid.org/0000-0002-0585-5513

Y.-S. Chao https://orcid.org/0000-0001-7433-5987

K.-F. Chung https://orcid.org/0000-0003-3628-2567

### Data availability

All of the data that support the findings of this study are available in the main text or Supplementary Information.

## Supplementary materials

Supplementary material 1 List of 250 basionyms of Vietnamese pteridophytes Data type csv Explanation note List of 250 basionyms of Vietnamese pteridophytes with their type localities (both original and interpreted), depository herbaria, protologues, years of publication, voucher information, collection years, GPS coordinates (interpreted), and coordinate uncertainties. https://binary.pensoft.net/file/1659814This dataset is made available under the Open Database License (http://opendatacommons.org/licenses/odbl/1.0/). The Open Database License (ODbL) is a license agreement intended to allow users to freely share, modify, and use this Dataset while maintaining this same freedom for others, provided that the original source and author(s) are credited. Cheng-Wei Chen, Germinal Rouhan, Viet Dai Dang, Hong Truong Luu, Van Truong Do, Thi Ngan Lu, Yi-Shan Chao, Yao-Moan Huang, Kuo-Fang Chung
